# Organization and molecular evolution of a disease-resistance gene cluster in coffee trees

**DOI:** 10.1186/1471-2164-12-240

**Published:** 2011-05-16

**Authors:** Alessandra F Ribas, Alberto Cenci, Marie-Christine Combes, Hervé Etienne, Philippe Lashermes

**Affiliations:** 1 IRD - Institut de Recherche pour le Développement, UMR RPB (CIRAD, IRD, Université Montpellier II), BP 64501, 34394 Montpellier Cedex 5, France; 2 CIRAD-BIOS - Centre de Coopération Internationale en Recherche Agronomique pour le Développement - Département des Systèmes Biologiques, UMR RPB (CIRAD, IRD, Université Montpellier II), BP 64501, 34394 Montpellier Cedex 5, France

## Abstract

**Background:**

Most disease-resistance (R) genes in plants encode NBS-LRR proteins and belong to one of the largest and most variable gene families among plant genomes. However, the specific evolutionary routes of NBS-LRR encoding genes remain elusive. Recently in coffee tree (*Coffea arabica*), a region spanning the *S*_*H*_*3 *locus that confers resistance to coffee leaf rust, one of the most serious coffee diseases, was identified and characterized. Using comparative sequence analysis, the purpose of the present study was to gain insight into the genomic organization and evolution of the *S*_*H*_*3 *locus.

**Results:**

Sequence analysis of the *S*_*H*_*3 *region in three coffee genomes, E^a ^and C^a ^subgenomes from the allotetraploid *C. arabica *and C^c ^genome from the diploid *C. canephora*, revealed the presence of 5, 3 and 4 R genes in E^a^, C^a^, and C^c ^genomes, respectively. All these R-gene sequences appeared to be members of a CC-NBS-LRR (CNL) gene family that was only found at the *S*_*H*_*3 *locus in *C. arabica*. Furthermore, while homologs were found in several dicot species, comparative genomic analysis failed to find any CNL R-gene in the orthologous regions of other eudicot species. The orthology relationship among the *S*_*H*_*3*-CNL copies in the three analyzed genomes was determined and the duplication/deletion events that shaped the *S*_*H*_*3 *locus were traced back. Gene conversion events were detected between paralogs in all three genomes and also between the two sub-genomes of *C. arabica*. Significant positive selection was detected in the solvent-exposed residues of the *S*_*H*_*3*-CNL copies.

**Conclusion:**

The ancestral *S*_*H*_*3*-CNL copy was inserted in the *S*_*H*_*3 *locus after the divergence between Solanales and Rubiales lineages. Moreover, the origin of most of the *S*_*H*_*3*-CNL copies predates the divergence between *Coffea *species. The *S*_*H*_*3*-CNL family appeared to evolve following the birth-and-death model, since duplications and deletions were inferred in the evolution of the *S*_*H*_*3 *locus. Gene conversion between paralog members, inter-subgenome sequence exchanges and positive selection appear to be the major forces acting on the evolution of *S*_*H*_*3*-CNL in coffee trees.

## Background

In their natural environment, plants encounter a vast array of pathogenic microorganisms such as viruses, bacteria, oomycetes, fungi and nematodes. To defend themselves against infection by these pathogens, plants employ a network of intertwined mechanisms. One such line of defense is based on dominant disease resistance (R) genes that mediate resistance to pathogens possessing corresponding avirulence (Avr) genes [1]. The largest class of known R genes includes those that encode the nucleotide binding site (NBS) and the leucine-rich repeat (LRR) domains. The deduced NBS-LRR proteins can be subdivided in classes based on their amino-terminal features [2,3]. The most frequent classes possess a TIR domain with similarity to either the intracellular signaling domains of Drosophila Toll and the mammalian Interleukin-1 Receptor or a CC domain (coiled-coil) in the N-terminal and are named TNL (TIR-NBS-LRR) and CNL (CC-NBS-LRR), respectively [1,3,4]. Each domain of NBS-LRR protein is predicted to have a specific function. The NBS domain is suggested to have NTP-hydrolyzing activity (ATPase or GTPase, etc), regulating signal transduction through conformational changes [4,5]. The LRR domain contains tandemly arrayed repeats in the carboxy-terminal region of R-genes and its predicted biochemical function is to mediate protein-protein interaction. It was hypothesized and experimentally confirmed that the LRR domain is involved in the specific recognition of pathogen effectors [6-9]. Both TIR and CC domains are assumed to be involved in protein-protein interactions and signal transduction [10,11].

Genes encoding NBS-LRR protein represent one of the largest and most variable gene families found in plants, with most plant genomes containing several hundred family members. NBS-LRR genes are unevenly distributed in plant genomes and are mainly organized in multi-gene clusters [2,12-16]. Furthermore, results of nucleotide polymorphism analyses demonstrated extremely high levels of inter and intraspecific variation of NBS-LRR genes, which presumably evolved rapidly in response to changes in pathogen populations [16-18]. The clustered distribution of R-genes is assumed to provide a reservoir of genetic variation from which new pathogen specificity can evolve via gene duplication, unequal crossing-over, ectopic recombination or diversifying selection [19]. However, the specific evolutionary routes of NBS-LRR encoding genes remain elusive. Several comparative sequence analyses of R-gene clusters have been performed across haplotypes or related genomes in different plant species including *Arabidopsis *[20,21], wild potato [22], tomato [23-25], *Brassicaceae *[26], wheat [27], rice [28] soybean [29] and common bean [30]. Available data suggest that different R genes can follow strikingly different evolutionary trajectories. Kuang et al. [31,32] divided NBS-LRR-genes into two evolutionary categories: Type I includes genes whose evolution is accelerated by frequent sequence exchange among paralogs. Consequently, their sequences have chimeric structure and a clear allelic/orthologous relationship between different genotypes cannot be easily established. Type II includes slowly evolving genes whose sequence mainly evolves through the accumulation of amino acid substitution. Orthology relationships are highly conserved among accessions [33].

The evolutionary rate of each domain of individual NBS-LRR-encoding genes has been shown to be heterogeneous [31]. The NBS domain appears to be subject to purifying selection, whereas the LRR region tends to be highly variable [34]. Nucleotide polymorphisms found in the LRR region of *R *genes have been shown to be responsible for pathogen specificity [8]. In particular, codons encoding putative solvent-exposed residues in the LRR domain are hypervariable among different R proteins and show significantly elevated ratios of non-synonymous to synonymous substitutions, suggesting that the LRR domain is subject to positive selection for amino acid diversification [19,35-39].

Coffee is one of the world's most important agricultural commodities and is the main livelihood of more than 80 million people worldwide. Although the *Coffea *subgenus *Coffea *includes more than 95 species [40], commercial coffee production relies mainly on two related species: *Coffea arabica *L. and *C. canephora *Pierre, which account for 65% and 35% of world coffee production, respectively (International Coffee Organization, http://www.ico.org). *C. arabica *L. (2n = 4x = 44), the only polyploid species in the *Coffea *genus, is an allotetraploid containing two diploid subgenomes, C^a ^and E^a^, which originated from two different diploid species (2n = 2x = 22), *C. canephora *and *C. eugenioides*, respectively [41]. While diversification in the *Coffea *subgenus *Coffea *probably occurred in the second half of the Middle Pleistocene (450,000-100,000 years BP), it is most likely that the allopolyploid speciation of *C. arabica *took place in relatively recent times i.e. from historical times to 50,000 years ago [41,42].

Among the diseases affecting cultivated coffee, coffee leaf rust, caused by the obligate parasitic fungus *Hemileia vastatrix *Berk. & Br. (Uredinales), is one of the most serious diseases and greatly limits Arabica coffee production in almost all coffee growing countries around the world. Therefore, the development of coffee varieties resistant to coffee leaf rust has been a breeding objective of the highest priority in many countries [43]. A number of resistance genes to coffee leaf rust have been identified in the cultivated or wild *Coffea *gene pool. In particular, one resistance gene (i.e. *S*_*H*_*3 *resistance factor) has been successfully introgressed from *C. liberica *into agronomically important Arabica cultivars. In the last few years, the genetic and physical maps of the *S*_*H*_*3 *locus were completed [44-46]. Furthermore, using fluorescence *in situ *hybridization in *C. arabica*, the *S*_*H*_*3 *locus was located in a distal position on a chromosome belonging to the homeologous group 1 [47]. Recently, a region of 800 kb spanning the *S*_*H*_*3 *locus was sequenced and annotated [48]. Tandem arrays of CNL R genes were identified suggesting that the *S*_*H*_*3 *locus corresponds to a complex multi-gene cluster.

The purpose of the present study was to gain insight into the genomic organization and evolution of the *S*_*H*_*3 *R gene cluster in coffee. The agronomic importance of this locus as well as the recent origin and the perennial characteristic of coffee species make these objectives especially appealing. Sequences of the *S*_*H*_*3 *region in three different genomes, the C^a ^and E^a ^subgenomes of *C. arabica *and the C^c ^genome of *C. canephora *were analyzed to investigate the genomic organization and evolution of the *S*_*H*_*3 *locus. In addition, we performed comparative analyses of the identified NBS-LRR encoding sequences to identify the forces that drive evolution in the *S*_*H*_*3 *R gene cluster. Our results highlight the importance of intra and inter subgenomic gene conversion as an important evolutionary mechanism for the evolution of disease resistance genes.

## Results

### Organization of the *S*_*H*_*3 *R gene cluster

**Figure 1:**
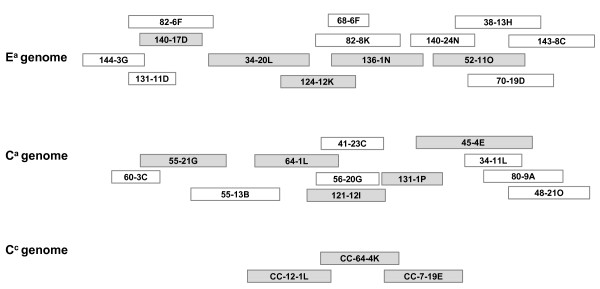
**BAC clone contigs spanning the *S***_***H***_***3 *locus**. BAC clones spanning the *S*_*H*_*3 *locus as previously contigued [46]. Sequenced BACs from the three genomes are indicated by gray boxes.

The sequences of a total of 13 BAC clones spanning the *S*_*H*_*3 *locus (Figure 1) in three coffee genomes (i.e. E^a ^and C^a ^sub-genome from *C. arabica *and C^c ^from *C. canephora*), were examined for the presence of R-genes using the previously determined annotation [48]. Depending on the genome concerned (E^a^, C^a^, C^c^), a total of 5, 3 and 4 R-genes were identified, respectively. These sequences shared more than 95% of identity. According to Chen et al. [49], a R gene family is defined when 60% identity is shared by members. The R-gene sequences detected in *S*_*H*_*3 *were therefore further considered as members of a unique R-gene coffee family. BLAST analysis of the non-redundant database (All non-redundant GenBank CDS translations + RefSeq Proteins + PDB + SwissProt + PIR + PRF) revealed high similarity with several R-genes belonging to the CNL class. While the highest identity was observed with the hypothetical CNL R protein in *Vitis vinifera *(36% identity) in *Ricinus communis *and *Glycine max *(35%), the most similar functionally characterized protein was the RPP8 gene from *Arabidopsis thaliana *that confers resistance to *Peronospora parasitica *[36] and shares 32% of identity and more than 50% of similarity. Among the species belonging to the subclass Asteridae, which includes *Coffea*, several proteins where found in *Solanum *spp. which show 30% of identity and 50% of similarity with the R-gene family found at the *S*_*H*_*3 *locus of *Coffea*.

**Figure 2:**
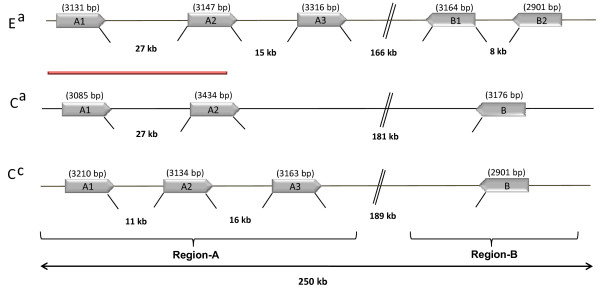
**Organization of *S***_***H***_***3-*CNL members in three coffee genomes**. E^a ^and C^a ^represent the sub-genomes from *C. arabica*, and C^c ^the genome from *C. canephora*. The red bar represents a 50 kb region where a homeologous non-reciprocal transposition event (HNRT) occurred between the two sub-genomes of *C. arabica *(unpublished data).

The CNL-like sequences identified in the *S*_*H*_*3 *R-gene cluster were distributed in two regions separated by more than 160 kb (Figure 2). In the first region (hereafter called region A) two or three copies were found in the same orientation. The other region (B) contained 1 or 2 copies repeated in tandem. Region A and B had opposing coding orientations. Orthology relationship among R-genes was established by comparing flanking sequences and each member was identified by the group letter followed by a number. A homeologous non-reciprocal transposition event (HNRT) occurred between the two genomes of *C. arabica *and involved a region of around 50 kb that includes the A1 and A2 members (unpublished data).

**Figure 3:**
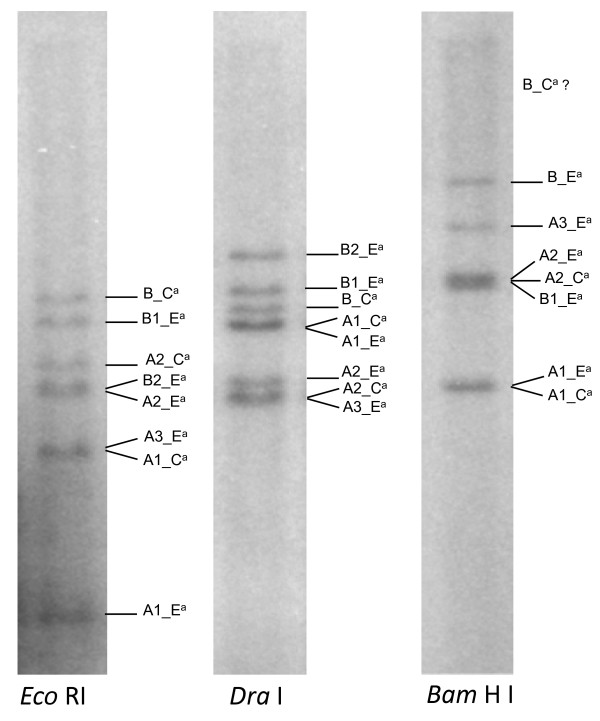
**Southern blot hybridization of genomic DNA of *C. Arabica***. DNA from the IAPAR-59 accession was digested with *Eco*RI, *Dra*I and *Bam*HI enzymes. The probe corresponded to the part of the NBS region that is highlighted by a frame in figure 6.

To test for the presence of a possible additional copy of the *S*_*H*_*3*-CNL in the Arabica coffee genome, Southern blot analysis was performed using a specific probe corresponding to a conserved part of the NBS region (Figure 3). Whatever the restriction enzyme used, only a limited number of hybridization bands was detected. Based on the restriction profiles predicted from sequence analysis of *C. arabica *cv. IAPAR-59 BAC, it was possible to assign all the bands to one of the eight members (five in the E^a ^genome and three in the C^a ^genome) present at the *S*_*H*_*3 *locus. No additional band was detected, suggesting that this family is only present at the *S*_*H*_*3 *locus in *C. arabica *cv. IAPAR-59. In fact, even if it is possible that additional hybridization fragments have size out of the detectable range, this should happened for all the three restriction enzymes and can be considered as a very improbable event.

**Figure 4:**
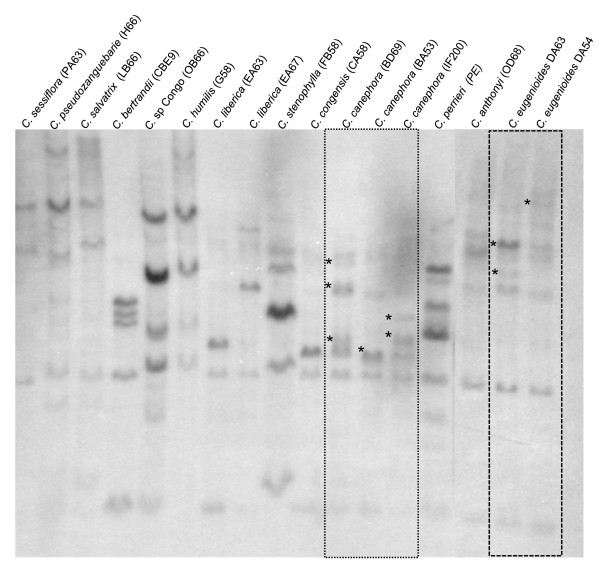
**Southern blot hybridization of genomic DNA from diploid coffee species**. *Eco*RI restricted DNA from diploid coffee species was probed with a NBS domain fragment. The stars indicate different band size among accessions of *C. canephora *and *C. eugenioides*.

The presence and number of members of this gene family in a panel of diploid coffee species were investigated by Southern blot analysis (Figure 4). While the *S*_*H*_*3*-CNL family was always present, the number of members ranged from three to eight depending on the species. Intra-specific variability was also observed for different accessions of *C. canephora *and *C. eugenioides*.

### Origin and evolution of the *S*_*H*_*3 *R gene cluster

**Figure 5:**
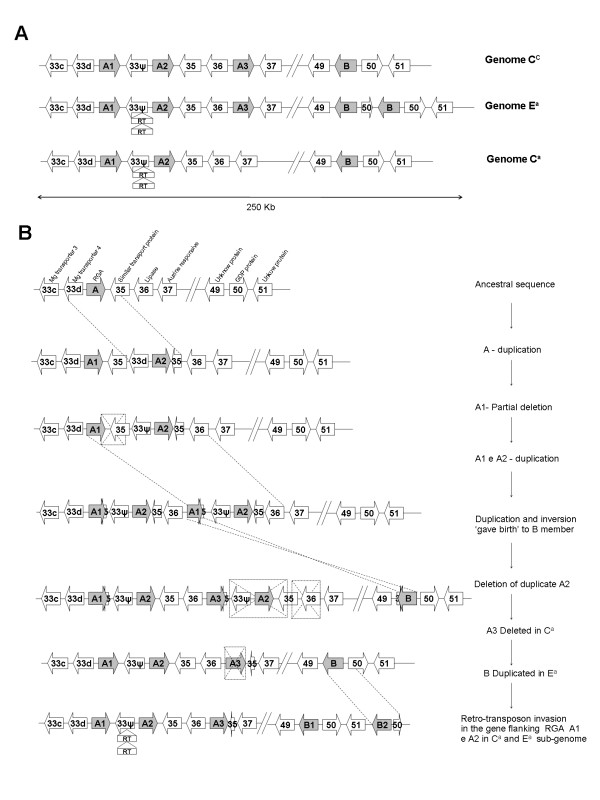
**Evolution of the *S***_***H***_***3 *locus in coffee species**. **A**. Current organization of the *S*_*H*_*3 *locus in *Coffea canephora *(C^c^) and *C. arabica *(sub-genome E^a ^and sub-genome C^a^). **B **- A model of the evolution of locus *S*_*H*_*3 *in coffee plants involving genome expansion and retraction by gene duplication and deletions. Gray arrows indicate members of the *S*_*H*_*3 *family. Open arrows indicate other non-R genes flanking R genes in the locus as numbered in [48]. Short arrows indicate truncated versions of corresponding genes.

To investigate the origin of the *S*_*H*_*3*-CNL genes present at locus *S*_*H*_*3 *we performed comparative analysis of the available sequences of three *Coffea *genomes and among the *S*_*H*_*3*-CNL copies including their flanking regions. Since members of the *S*_*H*_*3*-CNL family were found to be collinear in the comparisons of the three *Coffea *genomes (Figure 5A), we concluded that the observed organization of this locus predates the divergence between *C. eugenioides *and *C. canephora *lineages. The most parsimonious scenario for the evolution of this locus is illustrated in figure 5B. Two tandem duplications and several deletions shaped region A, whereas a distant duplication/insertion event gave birth to the *S*_*H*_*3*-CNL member(s) in region B.

Locus *S*_*H*_*3 *was compared with the putative orthologous region in the tomato genome (*Solanum lycopersicum*) which is, to date, the closest species to *Coffea *for which whole genome sequence is available (http://solgenomics.net). Micro-synteny was found between the coffee *S*_*H*_*3 *locus and two tomato genomic regions which shared 53.2 and 23.4% of the *Coffea *genes, respectively (data not shown), but no CNL genes were found in these regions of the tomato genome.

### Sequence characterization of the *S*_*H*_*3*-CNL family

**Figure 6:**
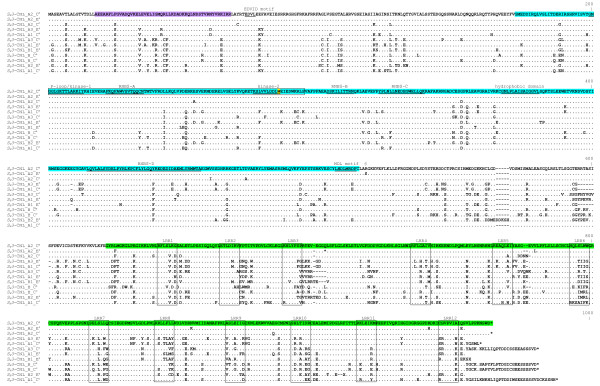
**Alignment of the predicted amino acid sequences from *S***_***H***_***3*-CNL members**. The coiled-coil, NBS and LRR domains are highlighted in lilac, blue and green, respectively. The motif EDVID [79] as well as the motifs P-loop/kinase 1, RNBS-A, kinase II, RNBS-B, RNBS-C, hydrophobic domain in NBS domain are underlined. The first sequence is shown in full, while for other proteins only amino acids that differ from the first one are indicated. A 8 bp deletions in B2_E^a ^and an 1 bp insertion in A2_E^a ^modifying the reading frame were disregarded. The xxLxLxx motif in the LRR domain is boxed, where L is any aliphatic amino acid and x is any amino acid. Gaps introduced at alignment are indicated by dashes, while asterisks indicate the presence of stop codons. NBS probe used in Southern hybridization is highlighted by a frame. The tryptophan residue (W), specific to the non-TIR-NBS-LRR class of plant disease R gene, located at the end of the kinase 2 motif [20], is highlighted in yellow.

The coding sequence of all *S*_*H*_*3*-CNL members is composed of two exons separated by an intron ranging from 157 to 272 nucleotides in length. The first exon spanned 1042 nt while the second exon extended from 1703 to 2003 nt (Table 1). The protein sequence extended from 915 to 1015 aa (Table 1). The protein sequence alignment of the identified 12 *S*_*H*_*3*-CNL members (eight from *C. arabica *and four from *C. canephora*) is shown in figure 6. *S*_*H*_*3*-CNL_A2_C^a ^was chosen as query to annotate protein domains. BLASTp analysis against the Pfam database predicted a NBS domain between positions 173 and 465 aa, while analysis of the Conserved Domain Database predicted the beginning of the LRR region at position 625 aa of the query protein. COILS analysis revealed a coiled-coil region located between position 17 and 56 aa, confirming that this family belongs to the CC sub-family of NBS-LRR genes (or non-TIR sub-family). The LRR region of all genes consists of 12 repeats ranging from 23 to 31 aa. These repeats are sufficiently different to ensure an unambiguous alignment of amino-acid sequences. A 8 bp deletions modified the reading frame of B2_E^a ^and induced an early stop codon after the 10th LRR; similarly, an 1 bp insertion in the A2_E^a ^made this member a pseudogene. Both INDEL modifying the reading frame were disregarded in figure 6 and in the following analyses.

**Table 1:** Exon, intron size (bp) and protein size (aa) of the *S*_*H*_*3*-CNL members identified in the three genomes analyzed.

Copy	genome	Exon 1	Intron	Exon 2	Protein (aa)
A_1_	E^a^	1042	272	1787	943
A_1_	C^a^	1042	255	1787	943
A_1_	C^c^	1042	270	1898	980
A_2_	E^a^	1042	270	1908	955
A_2_	C^a^	1042	270	1820	954
A_2_	C^c^	1042	269	1823	954
A_3_	E^a^	1042	268	2003	1015
A_3_	C^c^	1042	256	1865	969
B_1_	E^a^	1042	258	1865	969
B_2_	E^a^	1042	157	1703	915
B	C^a^	1042	260	1895	970
B	C^c^	1042	260	1874	972

### Cloning of *S*_*H*_*3-*CNL_A2 members from diploid species of coffee

To study interspecific diversity, the SH3-CNL_A2 member was selected at random for further analysis. The *S*_*H*_*3-*CNL_A2 member was cloned from six coffee species (*C. anthonyi, C sp*. Congo*, C. canephora, C. eugenioides, C. liberica, C. pseudozanguebarie*). The cloned fragments were around 4 kb in size. Their sequences were determined and compared with those from C^a^, E^a ^and C^c ^genomes.

### **Sequence diversity analysis of the ***S*_*H*_*3-*CNL **family**

Using the RDP3 software [50] and regardless of the method used for the analysis, significant traces of gene conversion were detected among the member of the *S*_*H*_*3*-CNL family, both in *C. arabica *and *C. canephora*. As an example, the conversions detected with the RDP method were reported in Table 2. Among the nine different gene conversions detected, two events involved inter subgenomic exchanges.

**Table 2:** Gene conversions detected among *S*_*H*_*3*-CNL members with the RDP method [50].

Sub-genome analyzed	*SH3*-CNL members	pValue	Begin	End	Length (Nc)
C^c^	A1_C^c ^× A2_C^c^	2.72 × 10^-14^	992	2249	1258
C^c^	A1_C^c ^× B_C^c^	6.57 × 10^-3^	1	977	977
C^a^	A2_C^a ^× A1_C^a^	2.13 × 10^-3^	2850	3049	200
C^a^	A2_C^a ^× A1_C^a^	4.15 × 10^-3^	1	579	579
E^a^	A1_E^a ^× A2_E^a^	9.46 × 10^-8^	1	1335	1335
E^a^	A3_E^a ^× B1_E^a^	7.63 × 10^-4^	218	1584	1367
E^a^	A2_E^a ^× A1_E^a^	7.62 × 10^-3^	2916	3054	139
C^a ^+ E^a^	B2_E^a ^× A1_C^a^	8.99 × 10^-14^	429	1232	804
C^a ^+ E^a^	A1_E^a ^× A2_C^a^	7.82 × 10^-8^	105	1335	1231

p Value (Bonferroni-corrected Karlin-Altschul); Begin, first nucleotide of the potential converted region; End, last nucleotide of the potential converted region; Length, length of the converted region (Nc).

**Figure 7:**
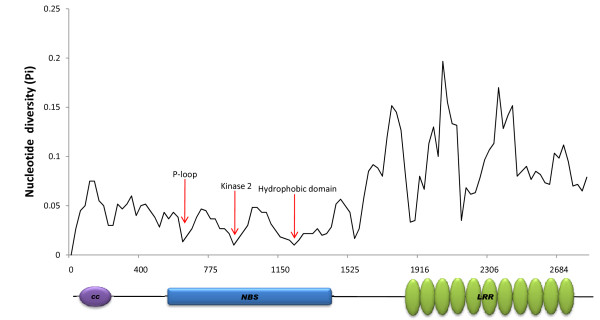
**Nucleotide diversity among *S***_***H***_***3*-CNL members from *C. canephora***. Nucleotide diversity (Pi) is the average number of nucleotide differences per site between two sequences calculated by DnaSP v.5. Nucleotide diversity was calculated using the sliding window method where a window (segment of DNA) is moved along the sequences step by step. The parameter is calculated in each window, and the value is assigned to the nucleotide at the midpoint of the window. Both the default values were used: window length of 100 sites, and step size of 25 sites (midpoint). The alignment gaps were not counted in the window length (or slide).

The DNA sp program (v.5) was used to estimate polymorphism among the four *S*_*H*_*3*-CNL members in the genome of *C. canephora *species (C^c^). The highest level of DNA polymorphism was detected in the LRR domain (π = 0.17, 0.20 and 0.15) while the most conserved regions were in the NBS domain, especially in the P-loop, Kinase 2 and hydrophobic domains (Figure 7).

To check the type of selection that acted on genes in the *S*_*H*_*3*-CNL family, the ratio between non-synonymous (Ka) and synonymous substitutions (Ks) was estimated using DNAsp v.5. The Ka/Ks substitution rate was calculated for each pair between ortholog and/or paralog members in *C. arabica *and *C. canephora *species. We also calculated the Ka/Ks between each pair of A2 members cloned from diploid coffee species together with A2 members from sequenced genomes (*C. arabica *and *C. canephora*). The analysis was performed on the complete coding sequence as well as on different domains (CC, NBS, LRR). Analysis also focused on codons encoding the solvent-exposed amino acids of the β-strand/β-turn motifs (x residues in xxLxLxx motifs) in the LRR domains.

**Figure 8:**
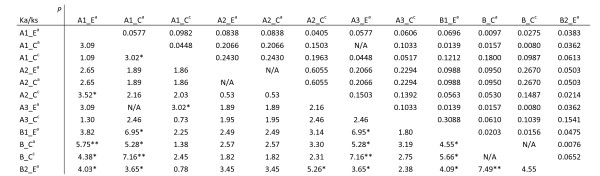
**Ka/Ks ratio in the solvent-exposed residue of *S***_***H***_***3*-CNL members**. The Ka/Ks ratio was calculated in the solvent-exposed residue of the LRR domain by pairwise comparison of *S*_*H*_*3*-CNL members. Values below the diagonal are the Ka/Ks ratio and values above are the probability, significance level for Ka > Ks indicated by * P < 0.05, ** P < 0.01. N/A = not applicable.

Strong evidence for positive selection (Ka/Ks > 1) was observed only for residues in xxLxLxx motifs. Among the 66 pair combinations between 12 BAC derived R-genes, 19, of which 16 involving B members, appeared under positive selection (Figure 8). When the same region was analyzed among orthologous A2 members, no significant Ka/Ks > 1 was found..

## Discussion

### **Organization and evolution of the ***S*_*H*_*3*-CNL **family**

Comparative analyses of R-gene clusters across different haplotypes or species demonstrated that the evolution of resistant genes is a dynamic process mostly involving duplication, deletion, sequence exchange, point mutation, diversified selection, recombination, gene conversion and retroelement insertion [21,23,24,28-30,32,33,51,52]. The cluster arrangement of R-genes represents an important reservoir of diversity and a source of genetic variation allowing the generation of novel resistance specificities via gene conversion, gene duplication, unequal crossing-over, ectopic recombination or diversifying selection [12,19]. To explore the organization and to characterize the mechanisms involved in the evolution of the *S*_*H*_3 locus, where a putative R-gene cluster was identified [48] a ~550 kb sequence was analyzed in three coffee genomes, E^a ^and C^a ^from *C. arabica *and C^c ^from *C. canephora*. Sequence analysis revealed the presence of a variable number of NBS-LRR genes belonging to the CC subclass at the *S*_*H*_3 locus. All these genes belong to the same family (hereafter called *S*_*H*_*3*-CNL family). Sequence analysis of regions flanking the *S*_*H*_*3*-CNL genes helped determine the orthology relationship among the copies in different genomes. At the same time, several traces of ancient duplications made it possible to trace back the duplication/deletion events which, consistently with the birth and death evolution model, shaped the *S*_*H*_*3 *locus from the most recent common ancestor of all *S*_*H*_*3*-CNL copies. Since the structure of the *S*_*H*_*3 *locus was well conserved in all three *Coffea *genomes analyzed, one can conclude that the origin of most of the *S*_*H*_*3*-CNL copies predates the divergence between *Coffea *species.

Homologs of *S*_*H*_*3*-CNL genes were found in several dicot species including *Solanum *spp., but comparative genomics failed to find a CNL R-gene in the orthologous regions of three Rosid species [48] and *Solanum lycopersicum*. Consequently, it can be suggested that the ancestral *S*_*H*_*3*-CNL copy was inserted in the *S*_*H*_*3 *locus after the divergence between *Solanum *and *Coffea *lineages.

In a similar study by David et al. [30] the authors compared the B4 locus of *Phaseolus vulgaris *(that has 26 CNL genes) with three sequenced legume genomes, *Medicago trunculata *(*Mt*), *Lotus japonicus *(*Lj*) and *Glycine max *(*Gm*). Their analysis revealed that conserved microsynteny existed among legumes species, except for the CNL sequences, which appeared to be completely absent in the corresponding regions of *Mt *and *Lj *and only a truncated CNL was found in syntenic regions of *Gm *[30]. They suggested that CNL were inserted in the ancestor of the B4 locus after *Mt, Lj*, and *Pv *diverged but before the divergence of *Pv *and *Gm *through an ectopic recombination event between non-homologous chromosomes. Phylogenetic analysis including those legume CNL sequences and all known *Pv *B4-CNL sequences supported this hypothesis [30].

Structural instability induced by repetitive mobile elements is one of the mechanisms that could lead to diversification into R gene families. The presence of very similar sequences increases the possibilities of mispairing during recombination, giving rise to unequal crossovers and interlocus gene conversions [19,53-55]. However, the edges of the duplications involved in the birth of new *S*_*H*_*3*-CNL copies were not related to mobile elements and mobile elements identified in the region did not appear to play a role in the structural evolution of the *S*_*H*_*3 *locus of *Coffea *species.

Gene conversion (i.e. the substitution of a portion of a gene sequence by the homologous sequence of another related gene) is more frequent among members of highly similar, tightly clustered families [56]. Gene conversion is a common phenomenon and it has been detected between paralogs in many R-gene clusters [9,21,35-37,57-62]. Sequence exchanges between different sub-genomes have previously been detected in a R1 resistance-gene cluster of one CNL subfamily in allohexaploid, *Solanum demissum *[22]. In that study, seven of the 17 sequence exchanges among R1 homologs occurred between different genomes. Two explanations were proposed: first, sequence exchanges among different haplotypes could be generated through gene conversions or alternatively, they might have occurred through recombination before speciation and have been conserved in *S. demissum *[22]. At the *S*_*H*_*3 *locus, gene conversion events were detected between paralogs in all three coffee genomes analyzed and also between members of the two sub-genomes of *C. arabica*.

Conversion events were detected between *S*_*H*_*3*-CNL members independently of their orientation (i.e. between members in region A and B).The inverted orientation of the loci might allow rare interlocus gene conversion or unequal exchange while minimizing the risk of gross chromosomal rearrangement [23]. The gene orientation in a cluster of NBS-LRR has also been studied in rice [12] and *A. thaliana *[56]. These studies demonstrated that conversion can occur between genes in the same or in opposite orientation, however conversion is more frequent in gene families arranged as direct repeats because they have higher similarity than those found in opposite orientation.

In addition, gene conversion was suggested to be more frequent in perennial than in annual plants. Yang et al.[18], compared the gene conversion events among NBS-encoding genes in two perennial and two annual plants. A total of 823 and 468 gene conversion events involving 299 and 187 NBS-encoding genes were detected in grapevine and poplar, respectively, while only 143 and 81 gene conversion events were detected in *Arabidopsis *and rice, respectively [18]. Since the long-generation time of woody species slows down the accumulation of evolutionary change, the authors suggested that an excess of recent duplications and a higher conversion rate in grapevine and poplar could generate novel resistance profiles to compensate for life history traits. According to Kuang et al. [31,32]*S*_*H*_*3*-CNL members should be classified as evolutionary type I (fast evolving genes) since several conversion events were detected between members.

### Effect of selection on molecular evolution of the *S*_*H*_*3*-CNL family

Natural selection influences the molecular evolution of sequences by increasing or reducing the fixation probability of a given mutation which, respectively, increases or reduces the fitness of the individuals carrying it.

The effect of natural selection on a gene sequence can be investigated by analyzing nucleotide substitutions that occurred between two variants of this gene. Since synonymous substitutions (i.e. nucleotide substitutions that do not change the amino acid sequence) are supposed to not modify the phenotype, their accumulation is considered not to be influenced by natural selection. Conversely, non-synonymous substitutions (nucleotide substitutions that modify the coded amino acid) could increase, reduce, or not influence the fitness of the individuals carrying it; consequently, their accumulation could be influenced by natural selection. The ratio of non-synonymous (Ka) to synonymous (Ks) substitution rates could be used to infer the effect of natural selection of a given gene or a part of it. When Ka and Ks have similar values (Ka/Ks ≈ 1), one could infer a neutral effect of selection; when Ka is significantly lower than Ks (0 < Ka/Ks < 1), it could be deduced that the selection purges the gene sequence of most non-synonymous substitutions (purifying selection); finally, when Ka is significantly higher than Ks (Ka/Ks > 1), the selection is assumed to favor fixation of new variants (positive or diversifying selection) [63,64].

In many NBS-LRR genes, analysis of corresponding proteins revealed high non-synonymous:synonymous substitution ratios in the leucine-rich (LRR) domain, mainly concentrated on the putative solvent-exposed residues, indicating that the LRR domain is subject to positive selection for amino acid diversification, [19,35,36,38,39,59,60,65-68]. These results are consistent with the observation that nucleotide polymorphisms found in the leucine-rich (LRR) region of *R *genes are often responsible for pathogen specificity [66].

In the *S*_*H*_*3*-CNL family, significant positive selection was only detected when the Ka/Ks analysis was focused on solvent-exposed residues (i.e. the x residues in xxLxLxx motif from LRR domain) most frequently among paralog members. Conversely, when larger regions were considered, the effect of natural selection was diluted and not detectable.

In the co-evolutionary arms race between hosts and their pathogens, genes involved in their interaction are expected to evolve under positive selection. The positive selection detected in the solvent-exposed residue of the *S*_*H*_*3*-CNL members could indicate involvement in recognition of pathogen attack.

## Conclusions

The *S*_*H*_*3*-CNL family appears to have evolved following the birth-and-death model, since duplications and deletions were inferred in the evolution of the *S*_*H*_*3 *locus. Gene conversion between paralog members from the same or different sub-genomes, and positive selection appear to be the major forces influencing the evolution of *S*_*H*_*3*-CNL in coffee trees.

## Materials and methods

### Plant material and DNA extraction

The cv. IAPAR 59 of *Coffea arabica *and six *Coffea *species were analyzed in this study: *C. canephora *(IF200), *C. anthonyi *(OD68)*, C sp*. Congo (OB66)*, C. eugenioides *(DA54)*, C. liberica *(EA67)*, C. pseudozanguebarie *(H66). Genomic DNA was isolated from leaves of greenhouse grown plants located at IRD (*Institut de Recherche pour le Développement*) Montpellier, France. Leaves were frozen in liquid nitrogen and DNA was extracted using a CTAB procedure [69] with modified extraction buffer (3% CTAB, 1.4 mM NACl, 100 mM Tris HCl, 20 mM EDTA, pH 0.8).

### BAC sequences

Several Bacterial Artificial Chromosome (BAC) clones spanning the *S*_*H*_*3 *locus were isolated from a *C. arabica *(IAPAR59) [70] and a *C. canephora *(HD-200-94) (unpublished data) libraries Based on fingerprint data and overlapping sequence analysis, BAC sequences were assembled in contigs specific to the *C. arabica *and *C. canephora *genomes (hereafter called C^c ^for *C. canephora *genome; E^a ^and C^a ^for "eugenioides" and "canephora" sub-genomes of *C. arabica*) (Lashermes et al. 2010). Gene annotation of the BACs was already available [48]. Sequences of the thirteen selected BACs were deposited to GeneBank [accession numbers, Genebank:GU123894 to GU123899 and HQ696507 to HQ696513].

### Primer design and cloning procedure

Orthologous specific primers to amplify A2 members of the *S*_*H*_*3*-CNL family from wild *Coffea *species were designed based on sequence alignments of A2 members in *C. canephora *and *C. arabica*: A2_Left: 5'-CCTTGATAAGAAACATGAATGAAATACACGA-3' and A2_right 5'-AAGGATAAATGAGAAGAACTACTGAGCCTG-3'. DNA amplification was performed with Expand™ 20Kb^plus ^PCR System (Roche Applied Science, Mannheim Germany). PCR were performed as follows: one cycle of 1 min at 95°C, 10 cycles of 10 sec at 94°C, 45 sec at 50°C, 5 min at 68°C followed by 20 cycles of 10 sec at 94°C, 45 sec at 50°C, 7 min at 68°C plus 10 sec per cycle, and final extension of 7 min at 68°C. A10 μl aliquot from each PCR amplification was analyzed by electrophoresis in a 1.2% agarose gel. The amplicons were gel-stained using Crystal violet and the DNA bands were purified using a S.N.A. P™ purification column (Invitrogen Carlsbad, CA). The PCR products were cloned into the pCR^®^-XL-TOPO^® ^kit from Invitrogen and chemically competent cells (Invitrogen Carlsbad, CA) according to the manufacturer's protocol. Eight colonies were randomly selected for screening. Colony PCR of eight random samples was used to select clones containing the complete amplicon. For this purpose, two new primers were designed to amplify the extremities of the genes in combination with the primers used to amplify the whole gene: 5'-CGACAGTGGGAACGAAACCC-3'combined with A2_Left and 5'-TGGAGGACCGGATCATGAACA-3' combined with de A2_RIGHT. The colony PCR was performed as follows: 10 min at 94°C, followed by 30 cycles of 30 sec at 94°C, 30 sec at 55°C, 4 min at 72°C and final extension of 10 min at 72°C. The colonies shown to contain the complete insert were transferred to 5 ml LB broth with 50 μg/ml kanamycin and incubated at 37°C overnight. Plasmid DNA was isolated using Promega Wizard^® ^*Plus *Minipreps DNA purification System (Promega Corporation, Madison, WI, USA) according to manufacturer's instructions. Two independent PCR and sequencing were carried out to ensure quality.

### Sequencing and analysis of cloned *S*_*H*_*3*-CNL members

Plasmid DNA was sequenced at Genome Express (Grenoble, France) using M13-universal- forward and reverse primers and five other internal primers were designed using the Primer3 program (Whitehead Institute, USA) to allow whole gene sequencing. INT1-L: 5-TCCATCGTCCAAGATACAGC-3, INT2-L: 5-TTTGTTGGGATGGAAGATGA-3, INT3-L: 5-GCTGGGAGTTGCTTCAAAAG-3, INT4-L: 5-TCGAATGTGGACAGCAGAAG-3, INT5-L: 5-GCCTTGGAGACACTTCCATC-3. The cloned sequence contigs were assembled using the Staden package [71]. The complete sequences of each clone were aligned using Bioedit v.7.0 [72].

### Southern blot analysis

Southern blot analysis was performed as follows: 20 μg of genomic DNA was extracted as described above and digested with a restriction enzyme (only *Eco*RI for the panel of diploid species *Eco*RI, *Dra*I and *Bam*HI for the *Coffea arabica *cv. IAPAR-59) and separated by agarose gel electrophoresis. The digested DNA was transferred to Hybond-N+ nylon membranes for Southern hybridization as described in Noir et al. [70]. *S*_*H*_*3*-CNL family specific probe was obtained by PCR amplification using primers designed on the NBS domain (left primer: 5'-CGGTCTCGGTAAGACCACTC-3'and right primer 5'-CCTCTGCAAATGGAAATGCT-3'). The amplified 516 bp fragment was labeled with [32P]-dATP according to the manufacturer's recommendations (Megaprime DNA Labelling Systems kit, Amersham) and used as probe in the hybridization experiment as described in Sambrook et al. [73].

### Motif predictions

SMART protein motif analyses (http://smart.embl-heidelberg.de) and Pfam database (http://pfam.sanger.ac.uk/search/sequence) were used to detect motifs in the *S*_*H*_*3*-CNL genes. COILS with a threshold of 0.9 was used to specifically detect CC domains [74].

### Gene Conversion

In order to check the possibility of conversion events among the the *S*_*H*_*3*-CNL members, alignments of sequences from *C. arabica *and *C. canephora *species were analyzed with the RDP3 software [50] using the default settings (but linear instead of circular sequences were selected, in general settings). The program uses simultaneously different recombination detection methods, including RDP and GeneConv [75], to both detect and characterize the recombination events that are evident within a sequence alignment without any prior user indication of a non-recombinant set of reference sequences [50]. Pairwise P values are assigned based on the comparison of each fragment with the maximum fragment length that is expected from the sequence pair by chance.

### Sequence evolution

Protein sequences were manually aligned with the BioEdit program. The amino acid sequence alignments were used to guide the alignments of nucleotides using MEGA version 4.1 [76]. Nucleotide diversity (π) was calculated by DnaSP v.5 [77] where each paralog was considered as an independent allele of population.

The *Ka/Ks *ratio was estimated by DnaSP v5.1 based on Nei and Gojobori's equation [78] for full-length CDS for specific domains: (CC, NBS, LRR), for the xxLxLxx motifs in the LRR domain, and for the solvent-exposed residues (i.e. only the x residues in the xxLxLxx motif). P values were calculated and the significance level was compared at 0.05 and 0.01%.

### Microsynteny Analysis

The search for putative gene orthologs of the genes at the *S*_*H*_*3 *locus was performed by TBLASTN analysis on the tomato genome sequence (*Solanum lycopersicum*) available in Solanaceae Genome Network (http://www.sgn.cornell.edu).

## Authors' contributions

AFR carried out the molecular genetic studies, participated in the sequence alignment and drafted the manuscript. AC carried out the genomic analyses and helped to draft the manuscript. MC participated in the sequence alignment. HE participated in the design of the study. PL designed the study, and participated in its coordination. All authors read and approved the final manuscript.
